# Comparative analysis and evaluation of wild and cultivated Radix Fici Simplicissimae using an UHPLC-Q-Orbitrap mass spectrometry-based metabolomics approach

**DOI:** 10.1038/s41598-024-58078-8

**Published:** 2024-03-28

**Authors:** Kai-Xin Guo, Yan-Fang Li, Hui Tang, Hao-Yang Wei, Wei Zeng, Xiao-Cui Yang, Yan Luo, Xue-Hong Ke

**Affiliations:** 1 Guangzhou University of Chinese Medicine, Guangzhou, 510405 China; 2 Qingyuan Traditional Chinese Medicine Hospital, Qingyuan, 511500 China; 3 Experimental Center, The First Hospital Affiliated to Guangzhou University of Chinese Medicine, Guangzhou University of Chinese Medicine, Jichang Road No. 16, Guangzhou, 510405 China

**Keywords:** Discrimination, Metabolomics, Wild *Radix Fici Simplicissimae*, Cultivated *Radix Fici Simplicissimae*, UPLC-Q-Orbitrap HRMS, Mass spectrometry, Metabolomics

## Abstract

*Radix Fici Simplicissimae* (RFS) is widely studied, and is in demand for its value in medicines and food products, with increased scientific focus on its cultivation and breeding. We used ultra-high-performance liquid chromatography quadrupole-orbitrap mass spectrometry-based metabolomics to elucidate the similarities and differences in phytochemical compositions of wild *Radix Fici Simplicissimae* (WRFS) and cultivated *Radix Fici Simplicissimae* (CRFS). Untargeted metabolomic analysis was performed with multivariate statistical analysis and heat maps to identify the differences. Eighty one compounds were identified from WRFS and CRFS samples. Principal component analysis and orthogonal partial least squares discrimination analysis indicated that mass spectrometry could effectively distinguish WRFS from CRFS. Among these, 17 potential biomarkers with high metabolic contents could distinguish between the two varieties, including seven phenylpropanoids, three flavonoids, one flavonol, one alkaloid, one glycoside, and four organic acids. Notably, psoralen, apigenin, and bergapten, essential metabolites that play a substantial pharmacological role in RFS, are upregulated in WRFS. WRFS and CRFS are rich in phytochemicals and are similar in terms of the compounds they contain. These findings highlight the effects of different growth environments and drug varieties on secondary metabolite compositions and provide support for targeted breeding for improved CRFS varieties.

## Introduction

*Radix Fici Simplicissimae* (RFS), the dry root of *Ficus hirta* Vahl., often called Guangdong ginseng or Wuzhaolong, is a widely distributed mulberry plant, occurring in Guangdong, Fujian, and Guangxi, in China^1,2^. RFS is a common medicine used by minority nationalities in the Lingnan region, especially the Yao and Zhuang nationalities^3^. It was first recorded in the Natural Preparation of Raw Herbs, and its application has beneficial impacts on the spleen and lungs, qi and dampness, muscles, and collateral circulation. Some applications of RFS include spleen deficiency and edema, insufficient food and abdominal distension, limb fatigue and weakness, lung deficiency and phlegm asthma, belching, night sweats, rheumatism and pain, postpartum non-lactation, and bruising^4–6^.

RFS was included in the 1977 edition of the Chinese Pharmacopoeia, and is a primary medicinal material used in Gongyanping tablets in the Chinese Pharmacopoeia^7^. It is also included in the Quality Standard of Yao Medicinal Materials in the Guangxi Zhuang Autonomous Region (Volume I) (2013 edition)^8^. Recently, with increasing attention being paid by the state to develop minority medicines, Wild *Radix Fici Simplicissimae* (WRFS) has been widely studied and frequently applied as a genuine Yao medicine. The value of RFS in medicine and food products is also increasingly researched and medically applied. RFS resources are primarily wild, but intensive land use and mining have reduced the wild RFS resources despite high market demand, while RFS cultivation has increased^9^. Cai et al.^10^ and Huang et al.^11^ assessed psoralen content as the quality standard of RFS and found great differences in the quality of RFS produced in different regions of Guangdong Province. However, research is lacking on WRFS and Cultivated *Radix Fici Simplicissimae* (CRFS) using technologies such as mass spectrometry combined with chemical pattern recognition, limiting standard development of RFS formulations.

Among several primary metabolomics research technologies, nuclear magnetic resonance (NMR) and chromatography coupled with tandem mass spectrometry (MS) are the most used. MS is used to identify metabolites using rapid, sensitive, and selective qualitative and quantitative methods, and combined with effective sample pretreatment and chromatographic separation, has high sensitivity and specificity. Liquid chromatography–mass spectrometry (LC–MS) uses high-throughput MS screening technology combined with metabolite identification, elucidates relevant biomarkers, and effectively analyzes the product components. Metabolomics combined with stoichiometry is used to monitor changes in the chemical components of traditional Chinese medicines from different sources^12,13^, growth sites^14^, and processing methods^15,16^, and for quality control (QC) of traditional Chinese medicines^17–19^.

In the early stage, we established the RFS fingerprint and the detection method of main components by HPLC^20^. In the present study, we used ultra-high-performance liquid chromatography quadrupole-orbitrap mass spectrometry-based metabolomics to elucidate the similarities and differences in phytochemical compositions of WRFS and CRFS. We aimed to analyze the effects of different growth environments and drug varieties on secondary metabolites and provide insights for targeted breeding of improved CRFS varieties.

## Materials and methods

### Plant materials

**Figure 1:**
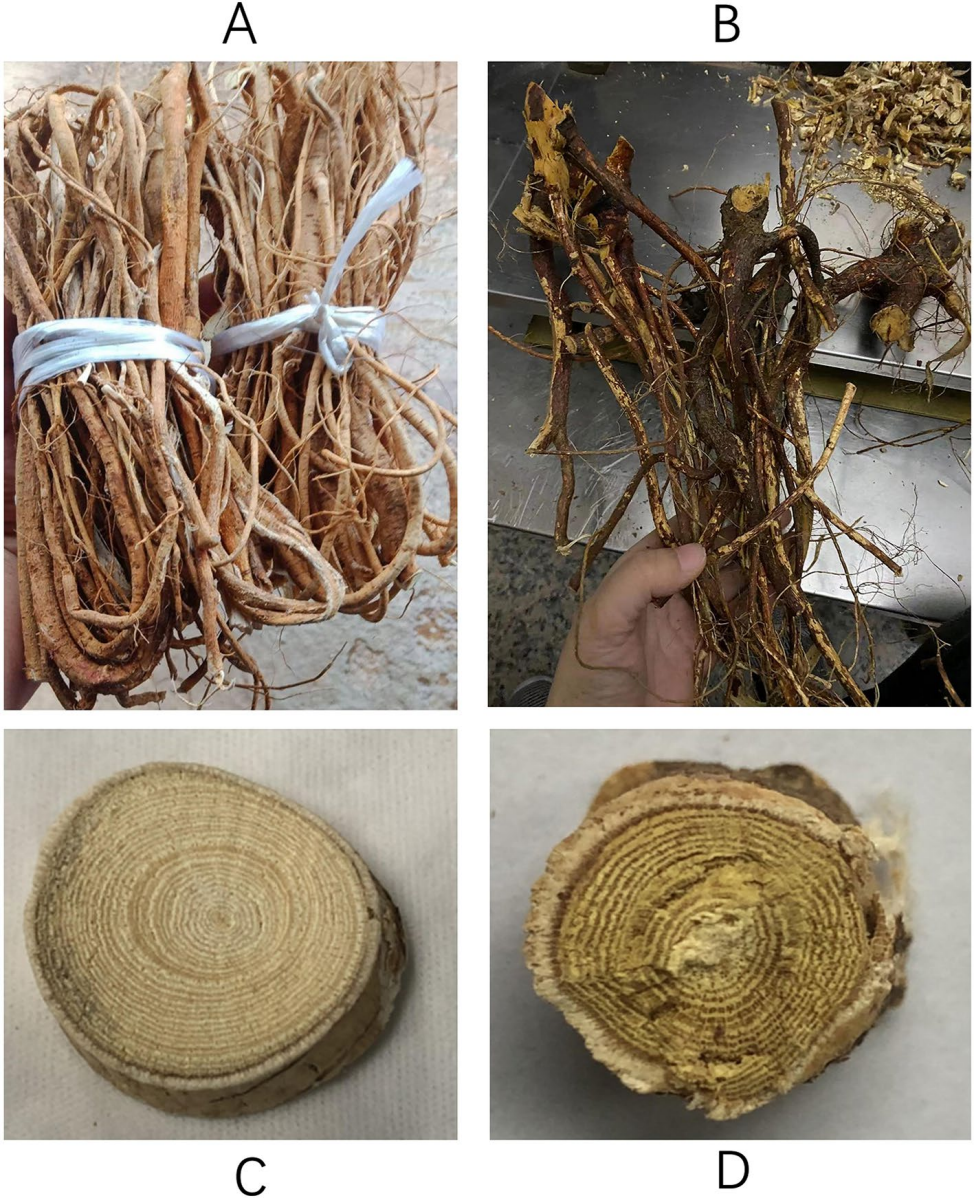
Phenotype of RFS : CRFS (wild *Radix Fici Simplicissimae)* (**A**,**C**) and WRFS (cultivated *Radix Fici Simplicissimae)* (**B**,**D**).

Qingyuan City is in the mountainous area of northern Guangdong Province and is a central residential area of the Yao nationality in China. In this study, 29 batches of RFS were harvested. Seventeen batches of WRFS samples were collected from Qingyuan City (Guangdong Province, China), and twelve CRFS samples were collected from hospitals and pharmacies (Table 1). Professor Yuan Xiaohong of Guangdong Provincial Hospital of Traditional Chinese Medicine identified all the medicinal materials. Among them, WRFS were provided by Qingyuan Traditional Chinese Medicine Hospital in May 2022, and CRFS were provided by Guangzhou First Affiliated Hospital of Traditional Chinese Medicine in June 2022. All samples were collected with the approvals from the respective authorities. The phenotypes of RFS are shown in Fig. 1.

**Table 1:** WRFS and CRFS samples from Guangdong province, China.

Species	Sample no.	Source	Collection time or batch number
WRFS	1	Lianshan County, Qingyuan City, Guangdong Province, China	October 2020
WRFS	2	Lianshan County, Qingyuan City, Guangdong Province, China	October 2020
WRFS	3	Lianshan County, Qingyuan City, Guangdong Province, China	October 2020
WRFS	4	Yingde County, Qingyuan City, Guangdong Province, China	October 2020
WRFS	5	Yingde County, Qingyuan City, Guangdong Province, China	October 2020
WRFS	6	Yingde County, Qingyuan City, Guangdong Province, China	October 2020
WRFS	7	Yingde County, Qingyuan City, Guangdong Province, China	December 2020
WRFS	8	Yingde County, Qingyuan City, Guangdong Province, China	December 2020
WRFS	9	Yingde County, Qingyuan City, Guangdong Province, China	December 2020
WRFS	10	Liannan County, Qingyuan City, Guangdong Province, China	January 2020
WRFS	11	Liannan County, Qingyuan City, Guangdong Province, China	January 2020
WRFS	12	Liannan County, Qingyuan City, Guangdong Province, China	April 2020
WRFS	13	Qingcheng County, Qingyuan City, Guangdong Province, China	April 2020
WRFS	14	Qingcheng County, Qingyuan City, Guangdong Province, China	May 2020
WRFS	15	Qingcheng County, Qingyuan City, Guangdong Province, China	May 2020
WRFS	16	Qingcheng County, Qingyuan City, Guangdong Province, China	December 2020
WRFS	17	Qingcheng County, Qingyuan City, Guangdong Province, China	December 2020
CRFS	18	Heyuan Jinyuan Green Life Co., Ltd. Jinlusheng Traditional Chinese Medicine Factory	220,101
CRFS	19	Guangdong Tiancheng Traditional Chinese Medicine Slices Co., Ltd	210,801
CRFS	20	Zhongshan Xianyitang Traditional Chinese Medicine Slices Co., Ltd	2,107,137
CRFS	21	Zhongshan Xianyitang Traditional Chinese Medicine Slices Co., Ltd	2,109,087
CRFS	22	Zhongshan Xianyitang Traditional Chinese Medicine Slices Co., Ltd	2,202,045
CRFS	23	Zhongshan Xianyitang Traditional Chinese Medicine Slices Co., Ltd	2,202,044
CRFS	24	Zhongshan Xianyitang Traditional Chinese Medicine Slices Co., Ltd	2,108,186
CRFS	25	Zhongshan Xianyitang Traditional Chinese Medicine Slices Co., Ltd	2,107,049
CRFS	26	Zhongshan Xianyitang Traditional Chinese Medicine Slices Co., Ltd	2,111,011
CRFS	27	Zhongshan Xianyitang Traditional Chinese Medicine Slices Co., Ltd	2,109,084
CRFS	28	Traditional Chinese Medicine Slice Factory of Guangdong Pharmaceutical Company	W2422311
CRFS	29	Traditional Chinese Medicine Slice Factory of Guangdong Pharmaceutical Company	W2422312

WFRS, Wild Radix Fici Simplicissimae; CRFS, Cultivated Radix Fici Simplicissimae.

#### Ethics statement

Collection of *Radix Fici Simplicissimae* in this research material conforms to and complies with the IUCN Policy Statement on Research Involving Species at Risk of Extinction and the Convention on the Trade in Endangered Species of Wild Fauna and Flora. In addition, according to the List of National Key Protected Wild Plants issued by the State Forestry and Grassland Bureau of China, *Radix Fici Simplicissimae*, the experimental material of this study, is not a national key protected wild plant nor an endangered plant species.

#### Sample preparation and extraction

The samples were ground and sieved (Chinese National Standard Sieve No. 3, R40/3 series) to obtain a homogeneous powder. Then, 0.1 g dried samples were added to a 5 mL volumetric flask, and 5 mL of 50% methanol was added. The mixture was left standing for 60 min and extracted using ultrasound (350 W, 35 kHz) (SK3300LH Ultrasonic Cleaner (Shanghai Kedao Ultrasonic Instrument Co., Ltd.)) for 60 min at 37 °C. Methanol (50%) was added to compensate for the loss in weight. The mixture was centrifuged (13,000 rpm; Thermo Legend Micro17R Centrifuge) for 10 min to obtain a clear solution. Additionally, a reference solution of psoralen and apigenin was prepared using the same method.

To ensure the suitability and stability consistency of MS analysis, a QC sample was prepared by pooling the same volume (10 µL) from every sample. In the entire worklist, one QC sample was inserted into every five test and analysis samples, and six QC injections were given to monitor the repeatability of the analysis. A volume of 3 µL was injected for each sample and QC. Metabolite extraction and detection repeatability were determined by overlapping the total ion flow diagram of MS detection and analysis of different QC samples.

### UPLC-Q-Orbitrap HRMS analysis

#### Liquid chromatography

Ultra-high-performance liquid chromatography quadrupole-orbitrap mass spectrometry (UPLC-Q-Orbitrap HRMS) analysis was performed on a Thermo QExactive mass spectrometer (Thermo Fisher Scientific, Waltham, MA, USA) equipped with a UPLC system through an electrospray ionization (ESI) interface. Samples were separated on a Thermo Hypersil Gold VANQUISH C18 (2.1 × 100 mm, 3 μm). The mobile phases were eluent A (0.1% formic acid in water, v/v) and eluent B (acetonitrile, v/v). The elution conditions applied were: 0–5 min, 5% B; 5–12 min, 25–80% B; 12–18 min, 80–99% B; 18–20 min, 99–5% B. The flow rate was 0.2 mL/min and sample injection volume was 3 µL. The column was maintained at 40 °C. Mass spectrometric grade formic acid, chromatographic grade methanol, and acetonitrile were purchased from Merck, Germany; ultrapure water and all other reagents were of analytical grade.

#### Mass spectrometry

The positive mode conditions were as follows: capillary voltage, 4.00 kV; carrier gas, nitrogen; sheath gas pressure, 3.5 MPa; auxiliary gas pressure, 1.0 MPa; capillary temperature: 320 °C; auxiliary gas heating temperature: 320 °C; primary resolution: 70,000. The negative mode conditions were identical to the positive mode conditions except for the capillary voltage (3.00 kV). The full scan mode was used, and positive and negative ions were detected simultaneously. The scanning range of the positive and negative ion spectra recorded by MS was 80–1200 m/z.

### Data analysis

#### Chemical component identification

For data collection, the samples were detected simultaneously in the first and second scanning modes under positive and negative ions, respectively, using UPLC-Q-Orbitrap HRMS (Thermo Fisher Scientific), and a total ion flow diagram was plotted. According to the pyrolysis spectrum detected in the electrostatic field orbital well analyzer, the accurate relative molecular weight, retention time, and multistage fragment ion information of the compound were obtained using a Compound Discoverer 3.2. The parameters were as follows: for 2D peak detection, 200 was set as the minimum peak area; for 3D peak detection, the peak intensities of low and high energy were set as > 1000 and > 200 counts, respectively; mass error in the range of ± 5 ppm was set for identified compounds; retention time in the range of ± 0.1 min was allowed to match the reference substance^21^. The predicted fragments generated from the structures were matched and identified against the mzCloud database and ChemSpider. Supporting information was obtained from relevant literature in databases such as PubMed.

#### Multivariate statistical analysis

The differences between WRFS and CRFS were explored using a metabolomics workflow. Multivariate statistical analysis was performed using SIMCA-P 14.0, and unsupervised principal component analysis (PCA) was used to obtain an initial understanding of the relationships between the data matrices. First, PCA was used to show pattern recognition and maximum variation to obtain an overview and classification. Second, the metabolite differences between different varieties of RFS and culture methods were detected using orthogonal projections to latent structures discriminant analysis (OPLS-DA) monitoring. OPLS-DA in ESI^+^ and ESI^−^ modes was performed to obtain the maximum separation between the CRFS and WRFS groups and to explore the potential biochemical markers contributing to the differences. S-plots were created to visualize the OPLS-DA predictive component loading to facilitate model interpretation. The corresponding variable importance for projection (VIP) was calculated in the OPLS-DA model, and VIP values were used to screen the different components. Metabolites with a VIP value of > 1 and a *p*-value of < 0.05 were considered potential markers. A heatmap was generated from these biochemical markers to visualize the variations in differential metabolites in the different groups, and metabolites with significant statistical differences among the classes were used to generate a heatmap in MetaboAnalyst4.0 (www.metaboanalyst.ca)^22^. We annotated the obtained differential metabolites using the Kyoto Encyclopedia of Genes and Genomes (KEGG) database and identified the corresponding pathways.

## Results and discussion

### Stability of the UPLC–MS/MS system

QC samples were used to evaluate the stability of the UPLC–MS/MS system. The curve overlaps between the metabolite detection and total ion current were high. The relative standard deviations (RSD) of the areas of all peaks were calculated, and the screening rates of the characteristic RSD < 30% in the positive and negative modes were 98.54% and 98.33%, respectively. These results suggest a high stability of the UPLC–MS/MS system throughout the experiment.

### Identity assignment and compound confirmation

#### Differential analysis in chemical composition between CRFS and WRFS

**Figure 2:**
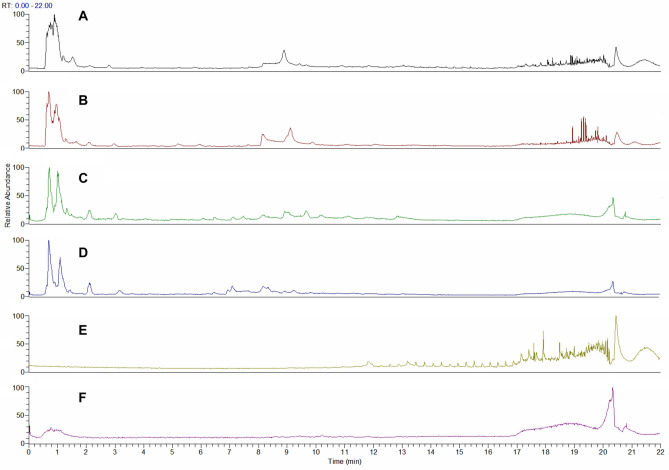
Base peak intensity (BPI) chromatograms of WRFS and CRFS in ESI^+^ and ESI^−^ modes: (**A**) WRFS in ESI^+^ mode; (**B**) CRFS in ESI^+^ mode; (**C**) WRFS in ESI^−^ mode; (**D**) CRFS in ESI^−^ mode; (**E**) Blank control in ESI^+^ mode (50% methanol solution); (**F**) Blank control in ESI^−^ mode (50% methanol solution).

The chemical profiles of WRFS and CRFS were analyzed using UPLC-Q-Orbitrap HRMS. A total of eighty one compounds were identified or tentatively characterized in ESI^+^ and ESI^−^ modes from WRFS and CRFS. Representative base peak intensity (BPI) chromatograms of the WRFS and CRFS are shown in Fig. 2.

**Figure 3:**
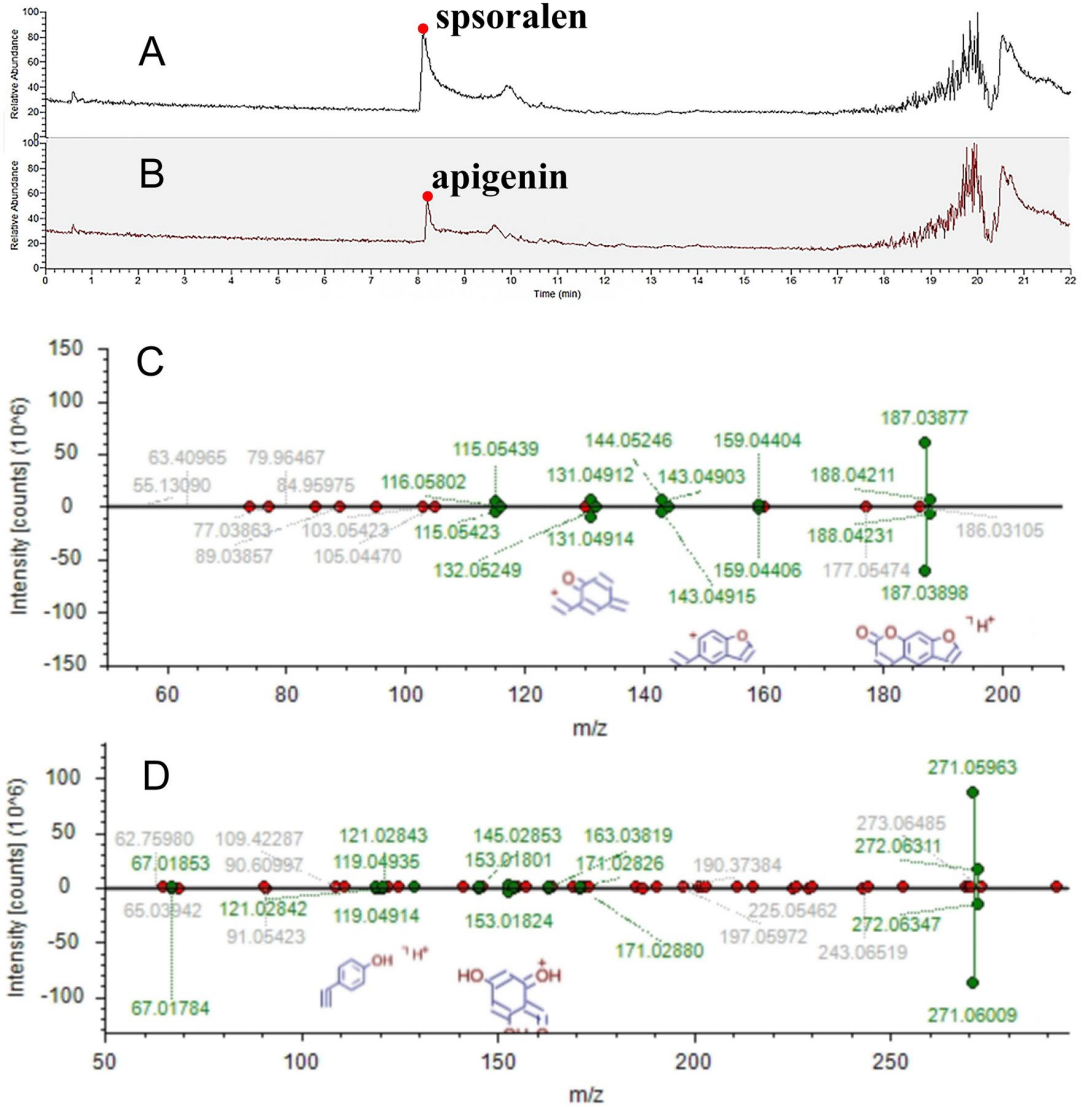
Ion chromatogram of spsoralen in ESI^+^ mode (**A**); Ion chromatogram of apigenin in ESI^+^ mode (**B**); Mass spectra of psoralen (**C**) and apigenin (**D**).

The similarity between the two BPI chromatograms was relatively high. Using Compound Discoverer 3.2 (Table 2), eighty one compounds were characterized from CRFS and WRFS, which were equivalent to [M + H]^+^ and [M−H]^−^ ions and were unambiguously or tentatively identified through a match with accurate molecular weights within a mass accuracy of < 5 ppm. Both types of RFS extracts were rich in compounds with various structural patterns, including flavonoids, coumarins, alkaloids, glycosides, organic acids, and organic acid esters. In addition, the ion chromatograms and mass spectra of psoralen and apigenin standards were compared, as shown in Fig. 3; the secondary fragment peaks were consistent with those of the corresponding compounds in Table 2, indicating the accuracy of compound identification by CD 3.2 software.

**Table 2:** UPLC-Q-Orbitrap HRMS-based determination of chemical composition of CRFS and WRFS cultivated in different ways.

No	RT (min)	Tentative identification of compound	Formula	Observed neutral mass (Da)	Observed m/z	Error (ppm)	Adducts	Main fragments via MS/MS
1	0.616	D-( +)-Proline	C_5_H_9_NO_2_	115.06313	116.07060	− 1.74	+ H	116.07036; 70.06546
2	0.707	Cytosine	C_4_H_5_N_3O_	111.04325	112.05054	− 0.11	+ H	112.05058
3	0.725	Betaine	C_5_H_11_NO_2_	117.0789	118.08626	− 0.68	+ H	118.08641
4	0.729	Trigonelline	C_7_H_7_NO_2_	137.04732	138.05495	− 2.64	+ H	138.05447
5	0.934	DL-Carnitine	C_7_H_15_NO_3_	161.10478	162.11247	− 2.55	+ H	162.11206; 103.03913
6	0.994	Guanine	C_5_H_5_N_5O_	151.04907	152.05669	− 2.26	+ H	152.05637
7	1.039	Nicotinic acid	C_6_H_5_NO_2_	123.03186	124.03930	− 1.33	+ H	124.03917
8	1.21	Hordenine	C_10_H_15_NO	165.11495	166.12264	− 2.48	+ H	166.12209; 121.06458
9	1.495	5-Hydroxymethyl-2-furaldehyde	C_6_H_6_O_3_	126.03152	127.03897	− 1.34	+ H	127.03873; 109.02834; 81.03376
10	1.506	l-Isoleucine	C_6_H_13_NO_2_	131.09436	132.10191	− 2.04	+ H	132.10146; 86.09663
11	1.676	2-(3-methoxyphenyl)acetamide	C_9_H_11_NO_2_	165.07861	166.08626	− 2.26	+ H	166.12196; 121.06451
12	2.092	l-Phenylalanine	C_9_H_11_NO_2_	165.07859	166.08626	− 2.33	+ H	166.08554; 120.08066
13	2.218	Pyrogallol	C_6_H_6_O_3_	126.03149	127.03897	− 1.6	+ H	127.03868; 109.02834; 81.03374
14	4.124	(1r,3R,4 s,5S)-4-{[(2E)-3-(3,4-dihydroxyphenyl)prop-2-enoyl]oxy}-1,3,5-trihydroxycyclohexane-1-carboxylic acid	C_16_H_18_O_9_	354.09409	355.10236	− 2.8	+ H	355.10049; 163.03877
15	4.71	Scopoletin	C_10_H_8_O_4_	192.04188	193.04954	− 1.99	+ H	193.04912; 178.02559
16	4.926	7-Hydroxycoumarine	C_9_H_6_O_3_	162.03126	163.03897	− 2.67	+ H	163.03860; 119.02550
17	5.225	Vitexin	C_21_H_2_0O_10_	432.1045	433.11292	− 2.66	+ H	433.11154; 313.06964; 283.05920
18	5.331	Orientin	C_21_H_2_0O_11_	448.09911	449.10784	− 3.23	+ H	449.10388; 329.06363; 299.05377
19	5.591	Vanillin	C_8_H_8_O_3_	152.04697	153.05462	− 2.43	+ H	153.05408; 125.05941; 111.04398; 93.03362
20	5.953	1,5-Anhydro-1-[5,7-dihydroxy-3-(4-hydroxyphenyl)-4-oxo-4H-chromen-8-yl]hexitol	C_21_H_2_0O_10_	432.10426	433.11292	− 3.21	+ H	433.10980; 313.06921; 283.05884; 337.06882
21	7.673	7,8-Dihydroxy-4-methyl coumarin	C_10_H_8_O_4_	192.04184	193.04954	− 2.19	+ H	193.04921; 175.03865
22	7.871	Luteolin	C_15_H_10_O_6_	286.0469	287.05501	− 2.92	+ H	287.05423
23	7.942	(2S)-2-(2-hydroxypropan-2-yl)-2H,3H,7H-furo[3,2-g]chromen-7-one	C_14_H_14_O_4_	246.08845	247.09649	− 3.07	+ H	247.09590; 229.08540; 175.03862
24	8.049	Eriodictyol	C_15_H_12_O_6_	288.06238	289.07066	− 3.5	+ H	289.06949; 163.03838; 153.01772
25	8.101	Naringenin	C_15_H_12_O_5_	272.06761	273.07575	− 3.16	+ H	273.07535; 153.01807; 147.04388
26	8.289	Psoralen	C_11_H_6_O_3_	186.03114	187.03897	− 2.98	+ H	187.03816; 143.04860; 131.04869
27	8.418	Apigenin	C_15_H_10_O_5_	270.05202	271.06010	− 2.96	+ H	271.05933
28	8.559	Apocynin	C_9_H_10_O_3_	166.06277	167.07027	− 1.32	+ H	167.06989
29	8.98	Diosmetin	C_16_H_12_O_6_	300.06233	301.07066	− 3.52	+ H	301.06989; 286.04636
30	9.716	Bergapten	C_12_H_8_O_4_	216.04168	217.04954	− 2.69	+ H	217.04901; 202.02550
31	10.198	Chrysin	C_15_H_10_O_4_	254.05736	255.06519	− 2.16	+ H	255.06454
32	10.201	Trioxsalen	C_14_H_12_O_3_	228.0781	229.08592	− 2.41	+ H	229.08533
33	11.24	Benzophenone	C_13_H_10_O	182.0727	183.08044	− 2.57	+ H	183.07977; 105.03344
34	11.884	Psoralidin	C_20_H_16_O_5_	336.0985	337.10705	− 3.79	+ H	337.10547
35	12.321	3,5-di-tert-Butyl-4-hydroxybenzaldehyde	C_15_H_22_O_2_	234.16121	235.16926	− 3.27	+ H	235.16846; 179.10611
36	12.338	5-hydroxy-2-(4-hydroxyphenyl)-8,8-dimethyl-4H,8H-pyrano[3,2-g]chromen-4-one	C_20_H_16_O_5_	336.09858	337.10705	− 3.56	+ H	337.10587; 283.05927
37	12.529	Cryptotanshinone	C_19_H_2_0O_3_	296.14038	297.14852	− 2.92	+ H	297.14764; 251.14246
38	0.675	D-(-)-Mannitol	C_6_H_14_O_6_	182.07827	181.07176	− 4.21	–H	181.07089; 101.02327; 89.02323; 71.01263
39	0.785	Gluconic acid	C_6_H_12_O_7_	196.05783	195.05103	− 2.42	–H	195.05052; 129.01845; 75.00767
40	0.803	D-(-)-Quinic acid	C_7_H_12_O_6_	192.06259	191.05611	− 4.16	–H	191.05530; 85.02831
41	0.846	d-Glucose 6-phosphate	C_6_H_13_O_9_P	260.02925	259.02244	− 1.8	–H	259.02206; 96.96844; 78.95784
42	1.078	α,α-Trehalose	C_12_H_22_O_11_	342.11581	341.10894	− 1.16	–H	341.10867; 179.05527; 119.03397; 113.02333; 101.02331; 89.02327; 71.01267; 59.01268
43	1.401	Citric acid	C_6_H_8_O_7_	192.02631	191.01973	− 3.62	–H	191.01921; 111.00783; 87.00772; 85.02847
44	2.082	3-Hydroxy-3-(methoxycarbonyl)pentanedioic acid	C_7_H_10_O_7_	206.04198	205.03538	− 3.25	–H	205.03505; 111.00767; 87.00760
45	2.621	3-[3-(beta-d-Glucopyranosyloxy)-2-hydroxyphenyl]propanoic acid	C_15_H_2_0O_9_	344.11042	343.10346	− 0.91	–H	343.10345; 181.04984; 163.03908; 137.05981
46	3.307	Quercetin	C_15_H_10_O_7_	302.04235	301.03538	− 0.99	–H	301.03494; 151.00244
47	3.64	Caffeic acid	C_9_H_8_O_4_	180.04157	179.03498	− 3.81	–H	179.03464; 135.04442
48	4.159	Catechin	C_15_H_14_O_6_	290.07881	289.07176	− 0.8	–H	289.07126; 245.08139
49	4.25	Chlorogenic acid	C_16_H_18_O_9_	354.09497	353.08781	− 0.33	–H	353.08823; 191.05576
50	4.397	2-(Acetylamino)hexanoic acid	C_8_H_15_NO_3_	173.10442	172.09792	− 4.45	–H	172.09688; 130.08630
51	4.601	Fraxetin	C_10_H_8_O_5_	208.03666	207.0299	− 2.49	–H	207.02928; 192.00574
52	4.76	4-Methylumbelliferone	C_10_H_8_O_3_	176.04658	175.04007	− 4.32	–H	175.03926
53	4.783	(1ξ)-1,5-Anhydro-1-[2-(3,4-dihydroxyphenyl)-5,7-dihydroxy-4-oxo-4H-chromen-8-yl]-d-galactitol	C_21_H_2_0O_11_	448.10026	447.09329	− 0.66	–H	447.09293; 357.06134; 327.05075; 299.05521; 298.04642; 297.04022
54	5.027	(9R,10R)-10-hydroxy-8,8-dimethyl-9-{[(2S,3R,4S,5S,6R)-3,4,5-trihydroxy-6-(hydroxymethyl)oxan-2-yl]oxy}-2H,8H,9H,10H-pyrano[2,3-h]chromen-2-one	C_20_H_24_O_10_	424.1365	423.12967	− 1.06	–H	423.12885; 179.05565; 89.02407; 71.01264; 59.01350
55	5.387	2-(acetylamino)-3-(1H-indol-3-yl)propanoic acid	C_13_H_14_N_2_O_3_	246.10008	245.09317	− 1.48	–H	245.09259; 203.08188; 116.03413; 116.04924; 98.02341; 74.02351; 70.02863; 58.02859
56	5.445	Cnidioside A	C_17_H_2_0O_9_	368.11046	367.10346	− 0.75	–H	367.10306; 205.04997; 161.05986
57	5.516	3-[4-(beta-d-Glucopyranosyloxy)-6-methoxy-1-benzofuran-5-yl]propanoic acid	C_18_H_22_O_10_	398.1212	397.11402	− 0.25	–H	397.11465; 235.06100; 191.07083; 176.04729
58	5.521	Suberic acid	C_8_H_14_O_4_	174.08848	173.08193	− 4.21	–H	173.08125; 111.08056
59	6.128	Isovanillic acid	C_8_H_8_O_4_	168.04145	167.03498	− 4.84	–H	167.03418; 152.01070
60	6.259	2,4,6-Trihydroxy-2-(4-hydroxybenzyl)-1-benzofuran-3(2H)-one	C_15_H_12_O_6_	288.06329	287.05611	− 0.36	–H	287.05649; 259.06131; 125.02356
61	6.904	Azelaic acid	C_9_H_16_O_4_	188.10412	187.09758	− 3.92	–H	187.09683; 125.09612
62	7.425	Diplosal acetate	C_16_H_12_O_6_	300.06303	299.05611	− 1.19	–H	137.02335
63	7.839	Ferulic acid	C_10_H_10_O_4_	194.05724	193.05063	− 3.47	–H	193.04990
64	8.196	Genistein	C_15_H_10_O_5_	270.0527	269.04555	− 0.44	–H	269.04538
65	8.546	Corchorifatty acid F	C_18_H_32_O_5_	328.22484	327.2177	− 0.42	–H	327.21732
66	9.094	Dodecanedioic acid	C_12_H_22_O_4_	230.15147	229.14453	− 1.46	–H	229.14404; 230.414699; 211.13297
67	9.233	Mycophenolic acid	C_17_H_2_0O_6_	320.1259	319.11871	− 0.28	–H	319.11868; 191.03410
68	9.318	Hispidulin	C_16_H_12_O_6_	300.06313	299.05611	− 0.87	–H	299.05582; 284.03232
69	9.792	(15Z)-9,12,13-Trihydroxy-15-octadecenoic acid	C_18_H_34_O_5_	330.24045	329.23335	− 0.52	–H	329.23294; 171.10173
70	10.085	Taurochenodeoxycholic acid	C_26_H_45_NO_6_S	499.29649	498.28948	− 0.54	–H	498.28958
71	10.247	Monobutyl phthalate	C_12_H_14_O_4_	222.08874	221.08193	− 2.12	–H	221.08136; 177.09140; 149.09613; 134.03627; 121.02839; 71.04902; 69.03339
72	10.749	Chrysin	C_15_H_10_O_4_	254.05765	253.05063	− 1	–H	253.05031
73	11.286	Asiatic acid	C_30_H_48_O_5_	488.34964	487.3429	− 1.09	–H	487.34369
74	12.666	N-(3-Chloro-4-morpholinophenyl)-6-oxo-1,4,5,6-tetrahydro-3-pyridazinecarboxamide	C_15_H_17_ClN_4_O_3_	336.09964	335.09164	2.15	–H	335.09204
75	12.871	16-Hydroxyhexadecanoic acid	C_16_H_32_O_3_	272.23496	271.22787	− 0.7	–H	271.22763
76	13.021	Oleic acid alkyne	C_18_H_30_O_2_	278.22431	277.2173	− 0.99	–H	277.21695
77	13.05	Dodecyl sulfate	C_12_H_26_O_4_S	266.15496	265.1479	− 0.84	–H	265.14767; 96.95897
78	13.303	(R)-3-Hydroxy myristic acid	C_14_H_28_O_3_	244.20354	243.19657	− 1.26	–H	243.19693; 59.01270
79	14.709	Ursolic acid	C_30_H_48_O_3_	456.35989	455.35307	− 0.99	–H	455.35260
80	15.503	Myristyl sulfate	C_14_H_30_O_4_S	294.18635	293.1792	− 0.43	–H	293.17935; 96.95897
81	15.738	Linoleic acid	C_18_H_32_O_2_	280.2402	279.23295	− 0.13	–H	279.23276

**Figure 4:**
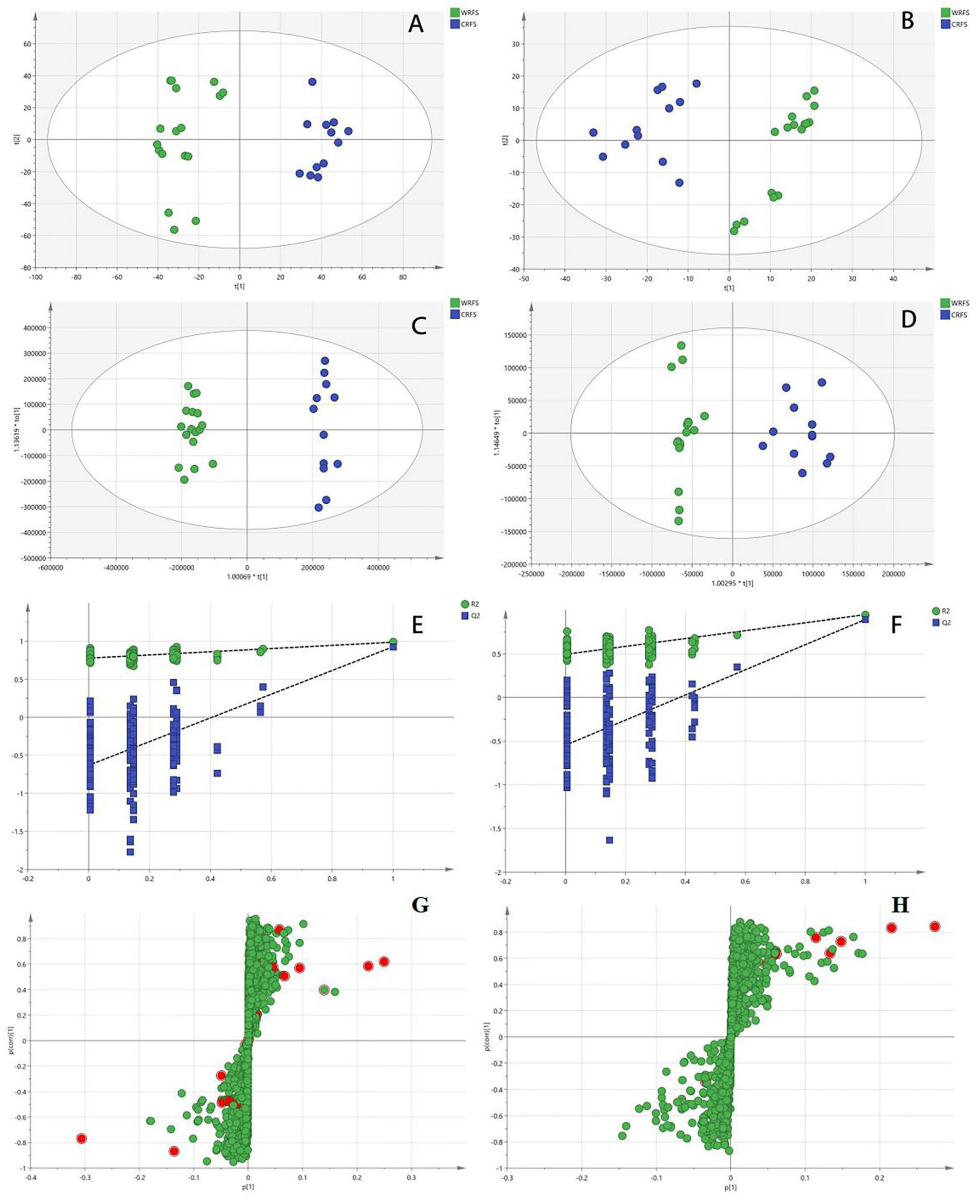
Principal component analysis (PCA) of WRFS and CRFS in ESI^+^ (**A**) and ESI^−^ (**B**) mode. OPLS-DA score plot with multivariate statistical analysis WRFS and CRFS in ESI^+^ (**C**) and ESI^−^ (**D**) mode. Cross-validation plot of OPLS-DA model with 200 permutation tests in ESI^+^ (E) and ESI^−^ (F) mode. OPLS-DA S-plot in ESI^+^ (**G**) and ESI^−^ (**H**) mode. (The red marked points in red of the S-plot graph G and H are potential chemical markers).

PCA is an important method for the dimensionality reduction of data and an unsupervised multivariate statistical pattern recognition method, and may be used to highlight specific samples from all data. The PCA score plots of WRFS and CRFS showed substantial aggregation separation (Fig. 4A,B). To evaluate the differences in RFS between different cultivation methods and to understand the variables responsible for sample separation, we determined the importance of the variables in the OPLS-DA scoring charts, S-charts, permutation tests, and projection values. OPLS-DA differs from PCA because it is a supervised discriminant analysis method with superior classification and prediction capabilities. OPLS-DA uses partial least squares regression to establish a relationship model between metabolite expression and sample categories to predict the sample categories. Therefore, the OPLS-DA method was used to determine the differences between WRFS and CRFS components. The WRFS samples were separated from CRFS samples in the OPLS-DA score plot (Fig. 4C,D), suggesting differences in biochemistry between WRFS and CRFS.

The data processed by Compound Discoverer 3.2 was imported into SIMCA-P 14.0 software, and unsupervised PCA was used to evaluate the classification trend and differences between groups. The R^2^X of the model in positive and negative ion mode was greater than 0.4 (0.492 and 0.522, respectively), indicating that the model was stable and reliable. Two hundred rounds of random permutations were performed to verify the established OPLS-DA model, indicating that the model was reliable (ESI^+^: R^2^X = 0.359, R^2^Y = 0.975 and Q^2^ = 0.926; ESI^−^: R^2^X = 0.333, R^2^Y = 0.945 and Q^2^ = 0.892, respectively) (Fig. 4E,F). Variables with VIP values of > 1 and *p* < 0.05 in the nonparametric test were considered potential biochemical markers between WRFS and CRFS. (Fig. 4G,H).

**Figure 5:**
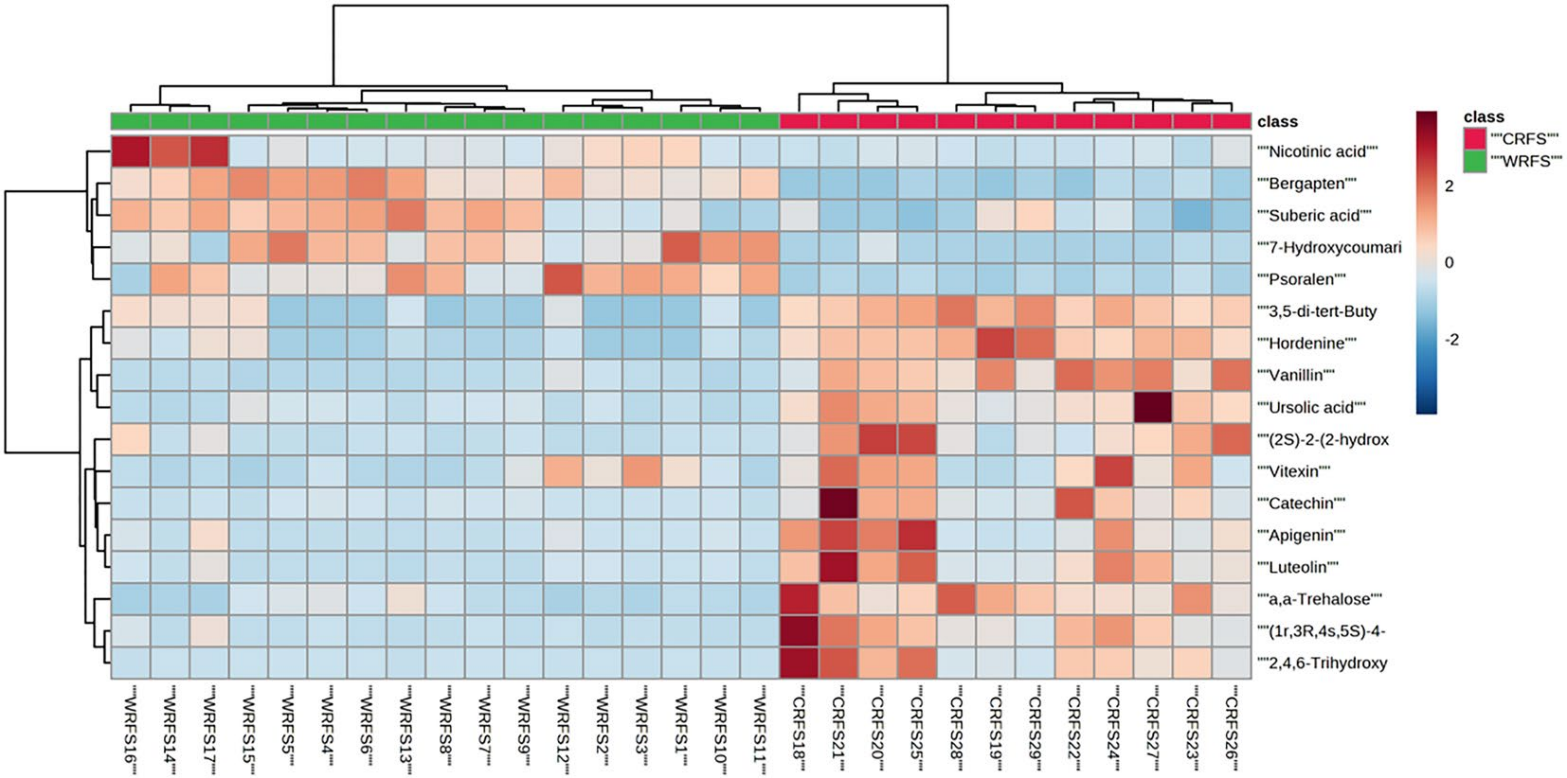
Heatmap of WRFS and CRFS metabolite content.

A heatmap was generated based on these markers to evaluate them systematically and intuitively (Fig. 5), and to show the strength of potential chemical markers between two samples. The close relationship of 17 potential markers is illustrated by combining the identification results as mentioned above. The samples were divided into two categories, WRFS and CRFS, and the results were consistent with those of the PCA. The 17 potential differential metabolites that could be considered as potential chemical markers for WRFS and CRFS were compounds 7, 8, 14, 16, 17, 19, 22, 23, 26, 27, 30, 35, 42, 48, 58, 60, and 79 (Table 3). The color indicates the signal strength of each metabolite; the darker the red, the greater the extent to which the metabolite appears above the average level of the sample, and blue indicates that the metabolite is at a lower level.

**Table 3:** Seventeen differential metabolites.

No.	Differential metabolite	Formula	VIP	Fold-change	Type	*p*-Value
7	Nicotinic acid	C_6_H_5_NO_2_	1.404	2.507	Up	1.101E−02
8	Hordenine	C_10_H_15_NO	4.194	0.205	Down	2.708E−07
14	(1r,3R,4 s,5S)-4-{[(2E)-3-(3,4-dihydroxyphenyl)prop-2-enoyl]oxy}-1,3,5-trihydroxycyclohexane-1-carboxylic acid	C_16_H_18_O_9_	1.243	0.109	Down	1.086E−03
16	7-Hydroxycoumarine	C_9_H_6_O_3_	1.610	13.176	Up	3.254E−06
17	Vitexin	C_21_H_2_0O_10_	1.071	0.439	Down	2.384E−02
19	Vanillin	C_8_H_8_O_3_	1.383	0.101	Down	1.563E−05
22	Luteolin	C_15_H_10_O_6_	1.595	0.109	Down	1.583E−03
23	(2S)-2-(2-hydroxypropan-2-yl)-2H,3H,7H-furo[3,2-g]chromen-7-one	C_14_H_14_O_4_	1.117	0.177	Down	3.224E−03
26	Psoralen	C_11_H_6_O_3_	22.854	7.535	Up	1.952E−06
27	Apigenin	C_15_H_10_O_5_	4.019	4.019	Up	6.356E−03
30	Bergapten	C_12_H_8_O_4_	10.046	8.541	Up	7.922E−10
35	3,5-di-tert-Butyl-4-hydroxybenzaldehyde	C_15_H_22_O_2_	1.034	0.271	Down	7.997E−09
42	α,α-Trehalose	C_12_H_22_O_11_	9.809	0.262	Down	8.921E−05
48	Catechin	C_15_H_14_O_6_	2.225	0.128	Down	7.591E−03
58	Suberic acid	C_8_H_14_O_4_	1.423	2.035	Up	1.594E−04
60	2,4,6-Trihydroxy-2-(4-hydroxybenzyl)-1-benzofuran-3(2H)-one	C_15_H_12_O_6_	5.414	0.042	Down	2.505E−03
79	Ursolic acid	C_30_H_48_O_3_	1.114	0.260	Down	1.515E−03

Up: compared with CRFS, the corresponding metabolite was upregulated in WRFS. Down: compared with CRFS, the corresponding metabolite was downregulated in WRFS.

### Kyoto Encyclopedia of Genes and Genomes analysis of differential metabolites

**Figure 6:**
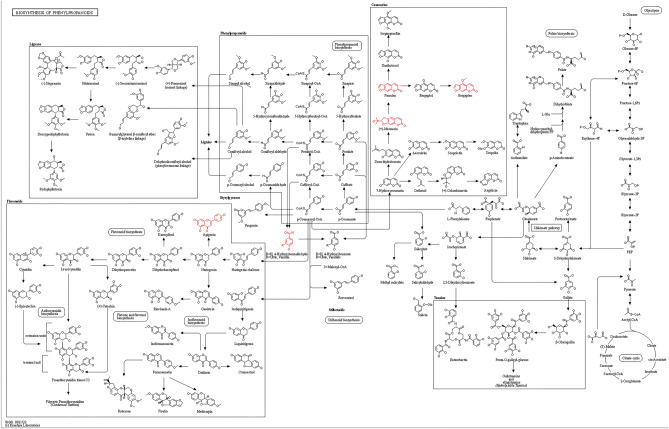
Phenylpropanoid biosynthesis. The compounds marked with red are differential metabolites belonging to phenylpropanes.

The KEGG database integrates genome, chemistry, and system function information and is a comprehensive dataset of metabolic pathway information^23–25^. The metabolic pathways are classified into different modules according to their functions, such as glycolysis, carbohydrate, TCA cycle, nucleoside and amino acid, organic compound and enzyme biodegradation, and other comprehensive metabolic pathways. Among the 17 differential metabolites, 14 were annotated to the KEGG database, 11 of which were annotated 29 times to KEGG pathways (Table 4). After removing duplication, 15 KEGG pathways were identified. Phenylpropanoid biosynthesis in the KEGG pathway is an example (Fig. 6).

**Table 4:** Categories of 14 differential metabolite-annotated KEGG pathways.

KEGG pathway	ID annotation	Number	Differential metabolite	Matching IDs
Biosynthesis of phenylpropanoids	map01061	5	Vanillin, apigenin, bergapten;(2S)-2-(2-hydroxypropan-2-yl)-2H,3H,7H-furo[3,2-g]chromen-7-one, psoralen	C00755|C01477|C01557|C09276|C09305
Flavonoid biosynthesis	map00941	3	Apigenin, vitexin, luteolin	C01460|C01477|C01514
Flavone and flavonol biosynthesis	map00944	2	Apigenin, luteolin	C01477|C01514
Biosynthesis of secondary metabolites	map01110	6	Apigenin, bergapten, psoralen, luteolin, nicotinic acid, 7-hydroxycoumarine	C00253|C01477|C01514|C01557|C05851|C09305
Nicotinate and nicotinamide metabolism	map00760	1	Nicotinic acid	C00253
Tropane, piperidine, and pyridine alkaloid biosynthesis	map00960	1	Nicotinic acid	C00253
Biosynthesis of alkaloids derived from ornithine, lysine, and nicotinic acid	map01064	1	Nicotinic acid	C00253
2,4-Dichlorobenzoate degradation	map00623	1	Vanillin	C00755
Starch and sucrose metabolism	map00500	1	α,α-Trehalose	C01083
Phosphotransferase system (PTS)	map02060	1	α,α-Trehalose	C01083
Phenylpropanoid biosynthesis	map00940	1	7-Hydroxycoumarine	C05851
Tyrosine metabolism	map00350	1	Hordenine	C06199
ABC transporters	map02010	1	α,α-Trehalose	C01083
Biosynthesis of alkaloids derived from the shikimate pathway	map01063	1	Vanillin	C00755
Metabolic pathways	map01100	3	Apigenin, luteolin, nicotinic acid	C00253|C01477|C01514

ID Annotation, ID of KEGG pathway; Number, the number of metabolites that can be annotated to the corresponding KEGG pathways; Matching IDs, Number of compounds in the KEGG pathway.

## Discussion

This study, a metabolomics study based on UPLC-Q-Orbitrap HRMS combined with multivariate statistical analysis revealed substantial differences in the compound compositions of WRFS and CRFS. The results of the identification and analysis of eighty one compounds showed distinct chemical profiles between WRFS and CRFS samples from different cultivation methods. Moreover, the identification results of these compounds in this study are consistent with those of Cheng Jun et al.^26,27^, which proves that RFS mainly contains phenylpropanoids, flavonoids, coumarins and other substances. Moreover, the chemical composition identification of RFS by Lao et al.^28^ and Zhao et al.^29^ showed that Vitexin, Vanillin, Luteolin, Psoralen, Apigenin, Bergapten, Ursolic acid and so on (17 potential differences between CRFS and WRFS) were consistent with our identification results. Using multivariate statistical analysis and a heatmap, WRFS and CRFS showed remarkable discrimination. Many markers exhibited different expression levels between the two samples. Psoralen, bergapten, and apigenin were upregulated in WRFS, and the content of these three active substances was much higher in WRFS than in CRFS. Many researchers have found that Psoralen, bergapten, and apigenin can be used as a quality marker of RFS, and it is the active ingredient with the highest content^30,31^.

*Radix Fici Simplicissimae*, one of ten famous medicines in Lingnan region, has been proven to play a role in protecting the liver, relieving inflammation, and having antioxidant and anti-cancer activities^32^. The ethanol extract of RFS can protect the liver of mice from alcohol-induced liver injury, probably by inducing and regulating downstream antioxidant factors, and also by suppressing the abnormal activation of CYP2E1 protein, reducing oxidative stress, and ultimately reducing the damage to the liver caused by alcohol^33^. Zhou Tiannong et al.^34^ found that compared with the control group, the water extract of RFS can significantly inhibit the increase of abdominal capillary diameter and improve the pain threshold of mice. It can also reduce the levels of alanine aminotransferase (ALT) and aspartate aminotransferase (AST) in mouse serum, which have good anti-inflammatory, analgesic, and liver protective effects. Deep research on the active components of RFS shows that it mainly consists of phenolic acids, terpenoids, flavonoids, coumarins, and phenolic acids^26,35^. Many scholars^36–39^ believe that the active components with a pharmacological effect that can be used as quality markers are psoralen, biflavonoids, and apigenin. Therefore, it should be studied as the main index. Psoralen, biflavonoids and apigenin have anti-tumor^40,41^, neuroprotective^42^, anti-inflammatory^43^, antioxidant and other pharmacological activities, which can be used to treat cancer, insomnia, Alzheimer's disease, rheumatoid arthritis, and aging. Psoralen and biflavonoids can prevent osteoporosis^44^, while apigenin can enhance immunity and prevent hypertension, arteriosclerosis, and cardiovascular and cerebrovascular diseases^45^. Because these different metabolites play an important role in health-related effects, these three components are very important for the quality evaluation of RFS. Our results show that the quality of WRFS is better than that of CRFS.

The main metabolic pathways that differ between WRFS and CRFS include primary and secondary metabolite biosynthesis. Psoralen, apigenin, and biflavonoids are annotated in multiple KEGG pathways related to phenylpropanoid biosynthesis, flavonoid biosynthesis, flavone and flavonol biosynthesis, and so on. Phenylpropanoid biosynthesis is an important metabolic process in humans, mainly involving the metabolism of amino acids such as phenylalanine and tyrosine. The process involves participation of various enzymes in catalyzing reactions to convert phenylalanine into other amino acids such as tyrosine. This biochemical process is crucial for the normal functioning of many physiological functions in the human body^46^. Flavonoids are an important branch of the phenylpropanoid metabolic pathway. The biosynthesis of flavonoids begins with phenylalanine, which is catalyzed by enzymes such as chalcone synthase to produce chalcone. Subsequently, the chalcone isomerizes into flavonoids, which then produces a variety of other flavonoid compounds, such as flavonols, isoflavones, and anthocyanins^47^. In addition, flavonoid and flavonols are two important components of flavonoids. Therefore, the results of this study provide clues for analyzing these metabolites and their metabolic networks in RFS. The variety and quantity of RFS collected in this study are limited, and its limitation should be attributed to the lack of sufficient sample size to support the research results, which can be expanded for further exploration.

This study showed that WRFS was superior to CRFS in quality, and explained the effects of different growth environments and drug varietie on secondary metabolites, and provides insights for further targeted breeding of improved CRFS varieties.

## Conclusion

In this study, a UPLC-Q-Orbitrap HRMS method was established and successfully applied to determine the component profiles of various RFS samples grown under different cultivation methods. Using multivariate statistical analysis and heat maps, WRFS and CRFS were shown to have significant differences. Psoralen, bergapten, and apigenin were significantly upregulated in WRFS compared to CRFS. Due to the important roles of these differential metabolites, our results indicate that the quality of WRFS is superior to that of CRFS, and this strategy will benefit the process of quality evaluation of RFS formulations.

## Data Availability

All data is available within the article or supplementary material.
